# Identification of expression profiles and prognostic value of RFCs in colorectal cancer

**DOI:** 10.1038/s41598-024-56361-2

**Published:** 2024-03-19

**Authors:** Md Misbah, Manoj Kumar, Abul Kalam Najmi, Mymoona Akhtar

**Affiliations:** 1 International Ph.D. Program in Medicine, College of Medicine, Taipei Medical University, Taipei, 110 Taiwan; 2 Centre for Translational and Clinical Research, School of Chemical and Life Sciences, Jamia Hamdard, New Delhi, 110062 India; 3 Department of Pharmacology, School of Pharmaceutical Education and Research, Jamia Hamdard, New Delhi, India; 4 Bioinformatics Infrastructure Facility, Jamia Hamdard, New Delhi, India; 5 Kusumraj Institute of Pharmacy, Bikram, Patna, Bihar India 801104; 6 Drug Design and Medicinal Chemistry Lab, Department of Pharmaceutical Chemistry, School of Pharmaceutical Education and Research, Jamia Hamdard, New Delhi, India

**Keywords:** Cancer genomics, Computational biology and bioinformatics, Cancer genetics

## Abstract

Colorectal cancer (CRC) ranks among the most prevalent cancers globally, with its incidence closely tied to DNA damage. The Replication Factor C (RFC) complexes comprises five protein subunits: RFC1, RFC2, RFC3, RFC4, and RFC5. These RFC complexes play crucial roles in DNA replication, repair pathways, activities post DNA damage, and ATP-dependent processes during DNA synthesis. However, the impact of RFC complexes proteins on CRC prognosis remains unclear. To explore this, we employed a computational analysis approach, utilizing platforms such as the DepMap portal, GEPIA, DAVID Bioinformatics for KEGG pathway analysis, Human Protein Atlas (HPA), STRING, and TIMER. Our results indicate that the mRNA levels of RFC1 and RFC5 were the least expressed among CRC cell lines compared to other RFC complex subunits. Notably, low RFC1 and RFC5 expression was correlated with poor prognosis in terms of CRC patients' overall survival (OS). Immunohistochemical results from the Human Protein Atlas demonstrated medium staining for RFC1, RFC2, and RFC5 in CRC tissues. Furthermore, the low expression of RFC1 and RFC5 showed a significant correlation with high expression levels of miR-26a-5p and miR-636, impacting cell proliferation through mismatch repair, DNA replication, and the nucleotide excision repair pathway. Although the precise functions of RFC1 in cancer are still unknown, our findings suggest that the small-molecule single target, CHEMBL430483, and multiple target molecules could be potential treatments for CRC. In conclusion, the elevated expression of miR-26a-5p and miR-636 targeting RFC1 and RFC5 expression holds promise as a potential biomarker for early-stage CRC detection. These insights provide novel directions and strategies for CRC therapies.

## Introduction

Colorectal cancer (CRC) is the world's third most common epithelial malignancy. According to the GLOBOCON database, around 147,950 individuals were diagnosed with CRC, and 53,200 patients succumbed to CRC in the United States in 2020^1^. Surgery is the primary intervention for CRC patients in the early stage of diagnosis; however, it proves ineffective in metastatic cases. About 20% of patients exhibit micro-metastasis or metastatic (m)CRC post-surgery^2,3^. Biomarkers like mitogen-activated protein kinase 1 (MAPK1), phosphatidylinositol 3-kinase (PI3K), and others are presently employed for CRC treatment^4,5^. An increasing number of biomarkers are being utilized for the diagnosis and selection of therapy for CRC patients^6^. Hence, identifying valuable biomarkers for patient identification is urgently needed in clinical practice.

The replication factor C (RFC) complexes function as clamp loaders responsible for loading and unloading proliferating cell nuclear antigen (PCNA) onto DNA, and play a role in various DNA replication and repair pathways, activities following DNA damage, and are also engaged in an ATP-dependent process during DNA synthesis^7–11^. The RFC family consists of five protein subunits, *RFC1, RFC2, RFC3, RFC4,* and *RFC5*^12^; which are strongly connected with tumor growth and metastasis^13^.

The Gene Expression Profiling Interactive Analysis (GEPIA), Human Protein Atlas (HPA) database, The Cancer Genome Atlas (TCGA) database, and Tumor Immune Estimation Resource (TIMER) were used for determining the gene expression level of RFCs^14^. Furthermore, protein–protein-interaction (PPI) and Kyoto Encyclopedia Genes and Genomes (KEGG) analysis aided in identifying the pathway for prognostic markers in CRC patients^15,16^.

MicroRNAs, short non-coding RNAs, can alter oncogene factors and mechanisms, by binding to the 3′-untranslated region (UTR) of their target messenger (m)RNAs leading to translational suppression. Onco-suppressor miRNAs can activate apoptosis, induce cell cycle arrest, impede cell viability, and tumor progression^17^. However, some previous studies identified, upregulated miR-26a-5p was shown to be a tumor suppressor oncomir in CRC^18^ and miR-636 was also identified onco-suppressor in many solid tumors^19^ The objective of this study was to identify treatment response biomarkers and comprehend the processes of the RFC family in colorectal cancer. In essence, the study aimed to discover microarray analysis predicting RFC's complex genes and underlying pathways in CRC patients.

## Results

### The expression level of RFCs in CRC using CCLE and GEPIA2

**Figure 1:**
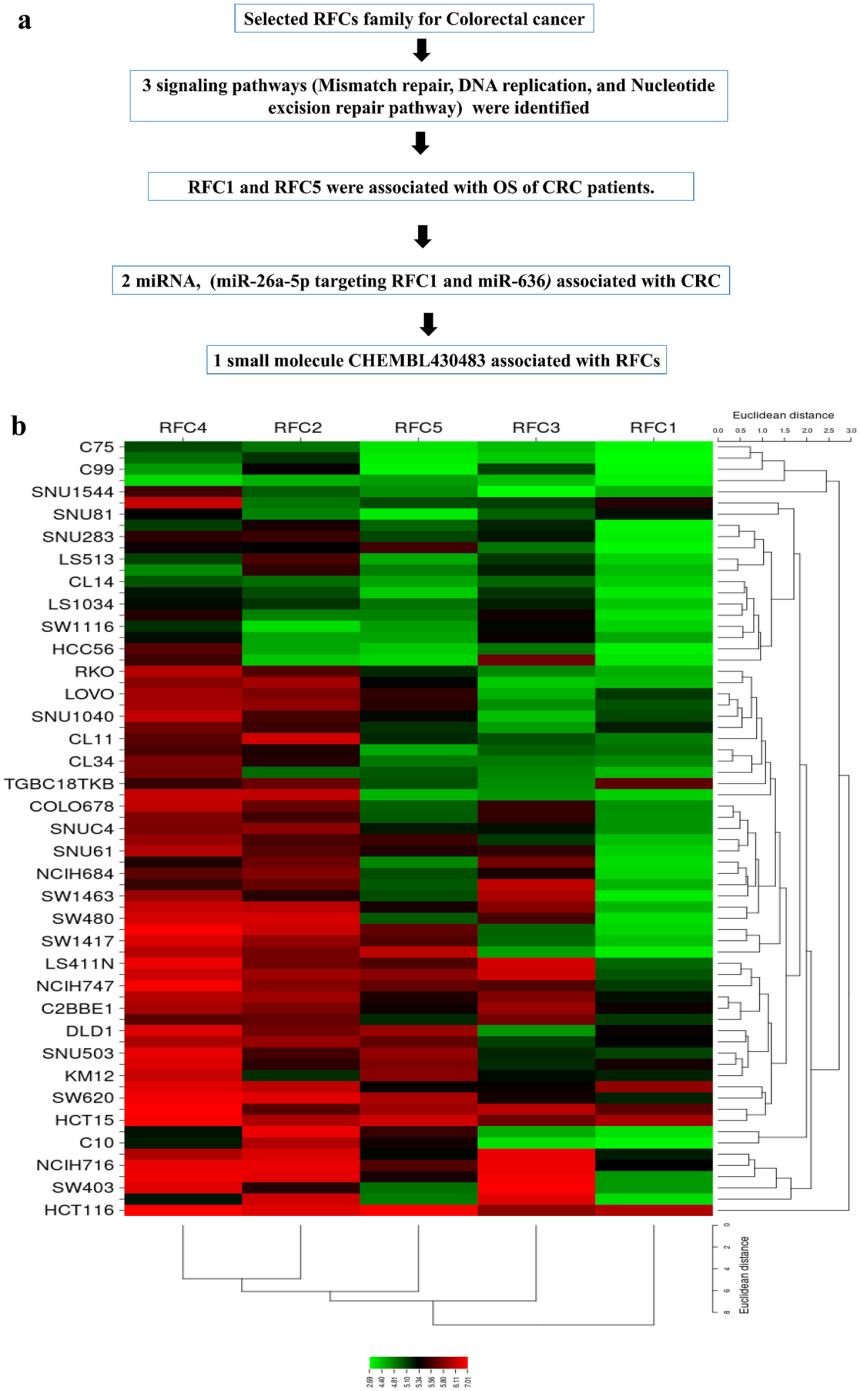
(**a**) Schematic diagram summarizing the study. (**b**) Heat map showing replication factor C subunits (RFC) gene mRNA expression in CRC cell lines (CCLEs) with red color signifying-overexpression, green color suggesting underexpression, and black colors show no expression.

**Figure 2:**
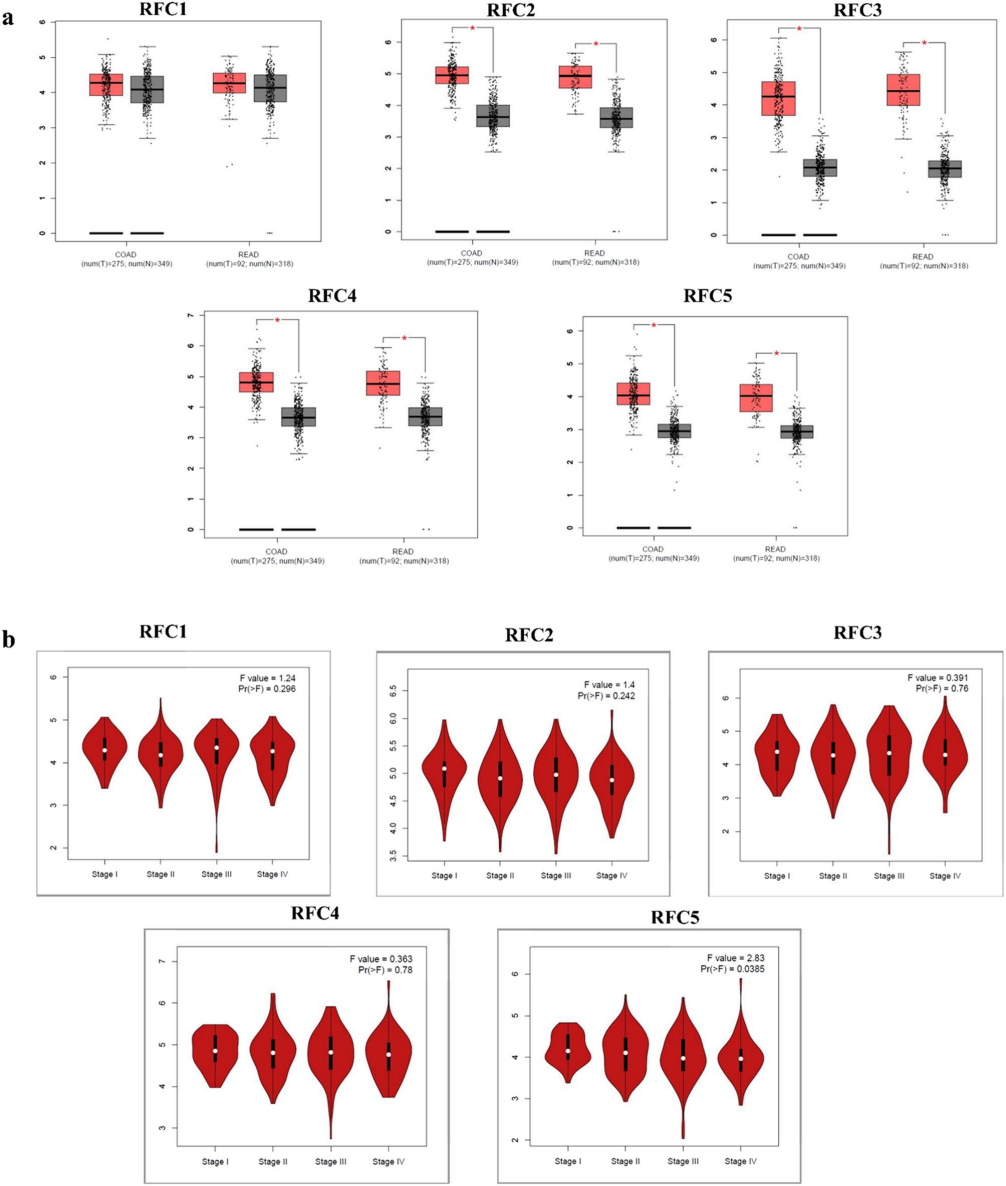
Gene expression profiles of (**a**) *RFC1*, *RFC2*, *RFC3*, *RFC4*, and *RFC5* in colorectal cancer (CRC). Boxplot showing transcriptional levels of Replication Factor C subunits gene (RFCs) in colon adenocarcinoma (COAD) (*n* = 275) vs. normal samples (*n* = 349) and rectal adenocarcinoma (READ) (*n* = 92) vs. normal tissues (*n* = 318) using the GEPIA web tool based on TCGA database. Black colors show transcriptional levels in normal tissues, while red colors show DEG transcriptional levels in COAD and READ tissues. A one-way ANOVA was used for the differential analysis with a statistically significant value of *P* < 0.05. (**b**) All stages of CRC are shown for cancer progression of the five RFCs. A violin plot shows different stages of cancer with log2 (transcripts per million (TPM) + 1) of genes in stages I to IV. A *t*-test was used with the statistically significant *p* < 0.05. The Pr(> F) < 0.05, followed by Student's *t*-test.

A schematic diagram showing a summary of all methodological techniques in a schematic diagram (Fig. 1a). The mRNA expression levels of RFC family members in CRC cell lines are presented through CCLE analysis (Fig. 1b). The heat map illustrates high mRNA expression of RFCs in certain cell lines, denoted by red and green colors. Additionally, we assessed RFC1, RFC2, RFC3, RFC4, and RFC5 expression in CRC using the GEPIA web tool analysis software. According to GEPIA analysis (Fig. S1 and Fig. 2), RFC2, RFC3, RFC4, and RFC5 displayed significant and distinct expressions in CRC. The boxplot comparing normal and colorectal adenocarcinoma indicated significant differences in RFC expression in CRC (Figs. S1A,B, Fig. 2A), except for RFC1, which showed no significant difference.

We further investigated the association between the expression levels of selected RFC genes and clinicopathological parameters. Figure 2B reveals that mRNA expression levels of RFC5 significantly differ across different tumor stages of CRC. However, the expression levels of RFC1, RFC2, RFC3, and RFC4 did not exhibit significant differences in various tumor stages. The RFC5 result aligns with a prior study indicating its relevance to cancer progression^20^.

### Genes associated with colorectal cancer patient’s survival and KEGG pathway analysis

**Figure. 3:**
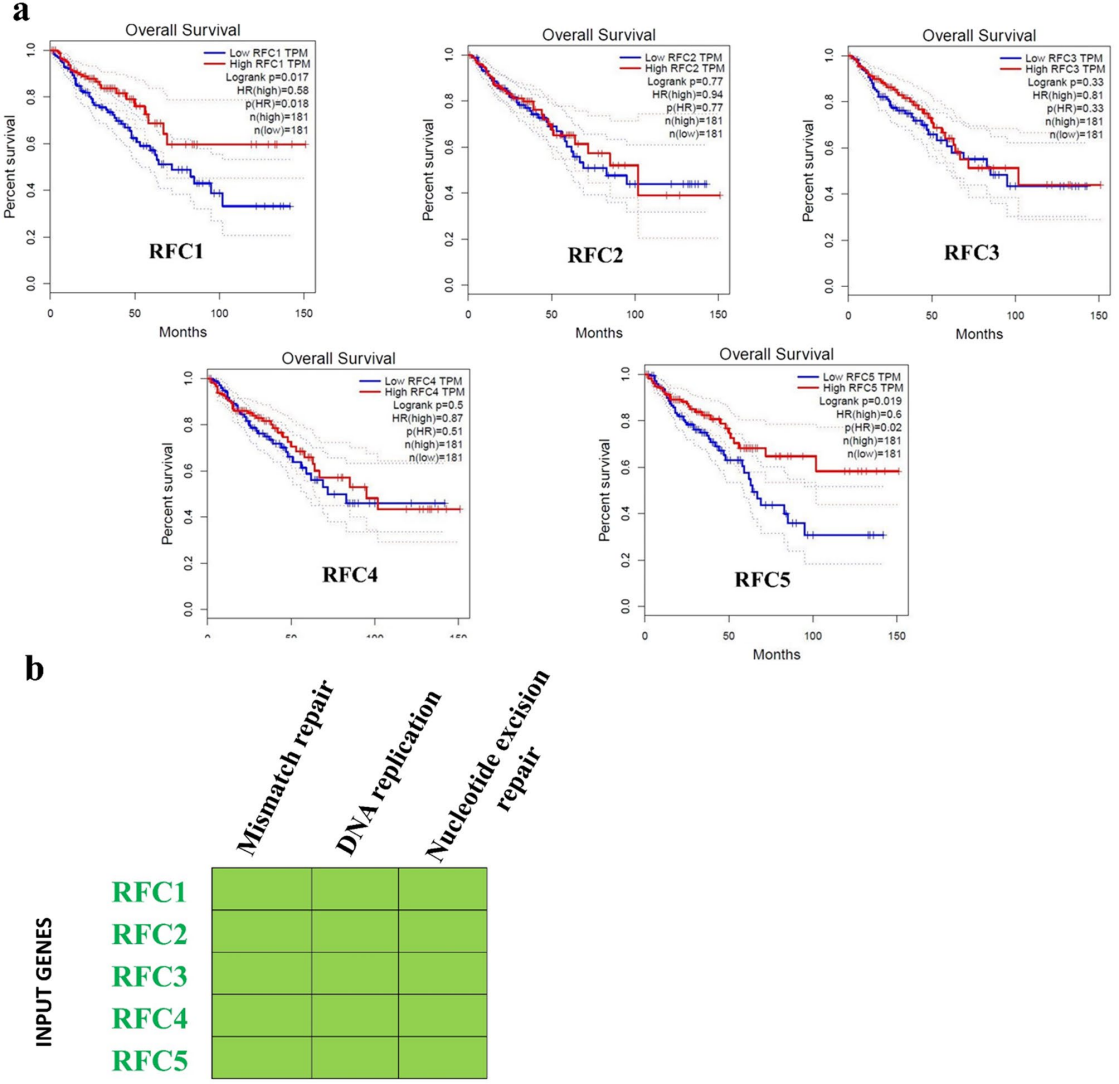
(**a**) Kaplan–Meier survival curves presenting the prognostic relationship between high and low expressions of replication factor C subunits (RFC) genes to overall survival (OS) in CRC patients using the GEPIA (TCGA) database patient samples. Survival curves were plotted using GEPIA online. Specific replication factor C subunits gene (RFC) expression levels were selected by the median value. Results are visually presented by Kaplan–Meier survival plots, and *p* values were calculated using log-rank statistics. Patient number (*n*) = 362, *p* = log-rank *p*-value with *p* < 0.05 considered significant. (**b**) Significant KEGG pathways and genes are involved. Gene enrichment analysis shows that KEGG pathways were significantly enriched in David pathway online analysis and genes involved in the pathways (the pathways are in order of their enrichment from left to right), *FDR* < 0.05).

Genes associated with colorectal cancer patient survival were analyzed. The GEPIA online web tool was utilized for survival analysis, revealing that low RFC1 and RFC5 levels were associated with a poor prognosis for CRC patients compared to RFC2, RFC3, and RFC4, as depicted in Fig. 3A.

The five RFC genes were subjected to KEGG pathway analysis using the DAVID online tool. Three KEGG pathways, namely mismatch repair, DNA replication, and nucleotide excision repair, were identified, and all reached statistical significance (FDR value < 0.25 and P-value < 0.05) for RFC1, RFC2, RFC3, RFC4, and RFC5 (Table 1). The expression pattern of candidates for mismatch repair, DNA replication, and nucleotide excision repair signaling are shown in Fig. 3B, where it shows that the five genes are downregulated.

**Table 1:** Enriched KEGG pathways.

KEGG pathway	Count	P-value	Genes	FDR
hsa03430:Mismatch repair	5	9.50E−11	*RFC5*, *RFC3*, *RFC4*, *RFC1*, *RFC2*	1.90E−10
hsa03030:DNA replication	5	6.32E−10	*RFC5*, *RFC3*, *RFC4*, *RFC1*, *RFC2*	6.32E−10
hsa03420:Nucleotide excision repair	5	1.91E−09	*RFC5*, *RFC3*, *RFC4*, *RFC1*, *RFC2*	1.91E−09

### The protein expression level of RFCs in CRC

**Figure 4:**
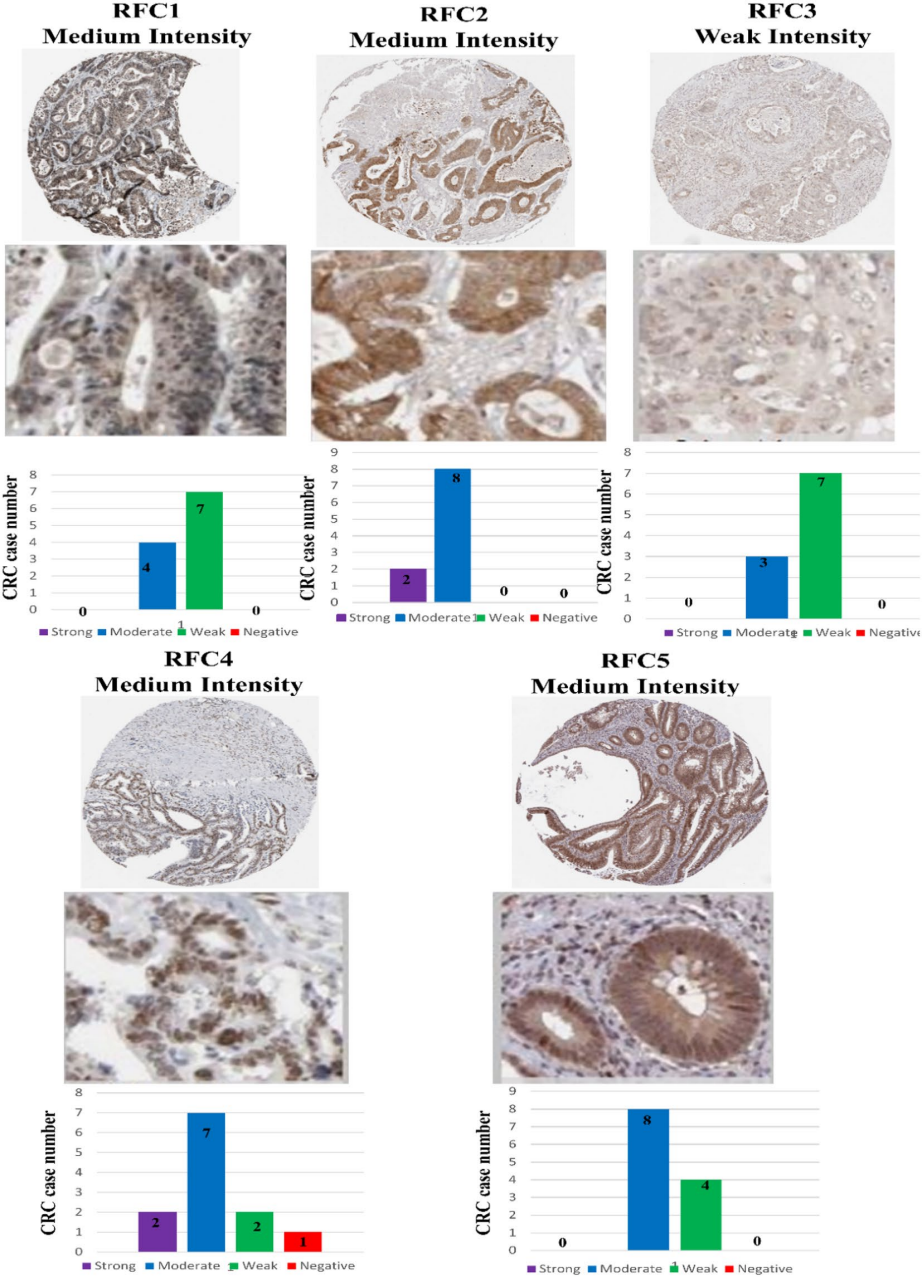
Protein expressions of five genes. The protein expression analysis used the HPA database of colorectal cancer (CRC) tissue samples. IHC images show the intensity and staining of replication factor C subunit genes (RFCs). Manual scoring of IHC data for staining intensity (negative, weak, moderate, or strong) and proportion of stained cells (> 75%, 25% ~ 75%, or 25%) as determined by the protein expression score.

The protein expression levels of five RFCs genes in 12 tissue samples from colorectal cancer patients were validated using the database Human Protein Atlas (HPA). IHC (immunohistochemistry) images of immunoreactivity expression in cancer specimens were examined (Fig. 4), and the staining intensity was manually scored.

The IHC image scoring categorized staining intensity as negative, weak, moderate, or strong based on the proportion of stained cells (> 75 percent, 25–75 percent, or < 25 percent, respectively), determining the protein expression score.

In colorectal cancer tissues, the staining levels of *RFC1*, *RFC2*, *RFC4*, and *RFC5* protein expression were modest. Overall, *RFC3* exhibited weak intensity while *RFC1* and *RFC5* displayed moderate intensity with > 75% quantity. In contrast, RFC2 and RFC4 demonstrated moderate intensity with a quantity ranging from 25 to 75%. Given that RFC1 and RFC5 exhibited intensity levels greater than 75%, they emerged as more promising biomarkers for colorectal cancer development.

### Identifying the potential miRNA target for the candidate’s genes

**Figure 5:**
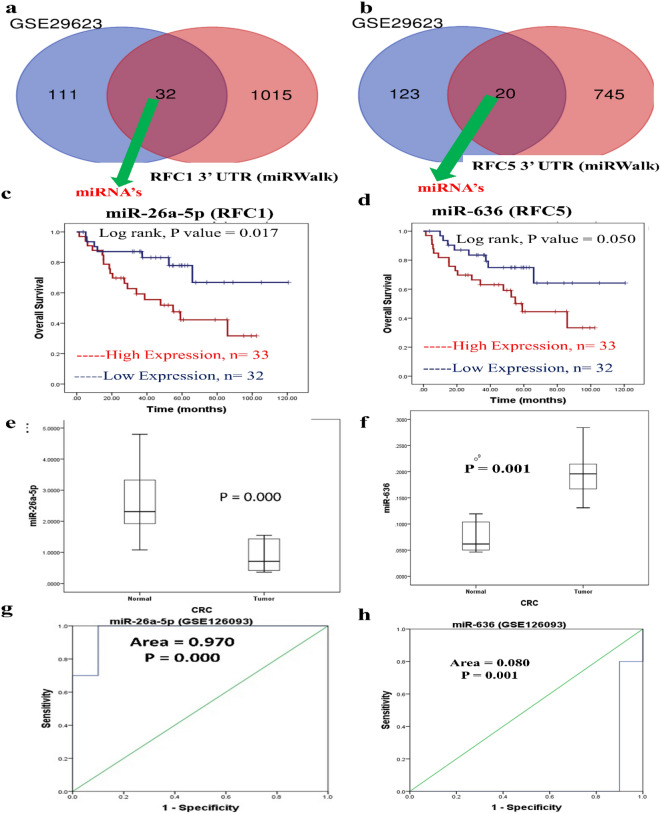
Venn-plot diagram showing miRNA targeting (**a**) *RFC1* 3′UTR and (**b**) *RFC5* 3′UTR in CRC identified from mirWalk vs GSE29623. Kaplan–Meier survival curves presenting prognostic relationships between high and low expressions of specific micro (mi)RNAs to overall survival (OS) using the GSE29623 database patient samples. (**c**) miR-26a-5p, and (**d**) miR-636 survival curves were plotted using SPSS 22.0. Specific miRNA expression levels were selected by the median value. Results are visually presented by Kaplan–Meier survival plots, and *P* values were calculated using log-rank statistics. Patient number (*n*) = 65, *p* = log-rank *P*-value, with *P* ≤ 0.05 considered significant. (**e**, **f**) GEO database analysis of normal vs. CRC tissues using the GSE126093 dataset. Expression levels miR-26a-5p, and miR-636 in colorectal cancer patients**.** *P* < 0.05 was considered significant. (**g**, **h**) ROC curve for normal vs. colorectal cancer (CRC) patients. MiR-26a-5p and miR-636 were considered significant.

To identify potential upstream regulators for those miRNAs, we employed MirWalk, webtools, and GSE29623 (CRC miRNA's) for miRNA prediction. The analysis revealed that 32 miRNAs were predicted to target RFC1, while 20 miRNAs were predicted to target RFC5 (Fig. 5A,B). Subsequently, we narrowed down the selection to 20 miRNAs for further analysis (Table S1).

### miRNAs associated with survival of patients with colorectal cancer

The overall survival analysis of targeted miRNA expression levels of CRC tissue samples was analyzed and validated using the GSE29623 database. A Kaplan–Meir plot was generated using statistical software SPSS version 22.0. The correlation of four miRNAs with the clinical outcome of CRC was further validated as shown in Fig. 5C and D. High expression of miR-26a-5p and miR-636 showed significant association (P = 0.024 and P = 0.050) with poor prognosis of CRC patients. However, the other seventeen miRNAs did not exhibit a statistically significant correlation (P > 0.05) with the overall survival of CRC patients (Table S2).

### Clinical validation of miR-26a-5p and miR-636 on CRC patients

The GEO dataset GSE126093 includes miRNA profiles from tissues of 20 CRC patients, with 10 CRC tissues and their corresponding normal-appearing tissues (NATs). Patients with tumor size > 5 cm, lymph node metastases (Lx group), stage III–IV, or metastases were higher than those in patients with < 5 cm tumor size, without lymph node metastases (L0), I–II stage or non-metastases, respectively^21^. The expression levels of miR-26a-5p were significantly lower in CRC patients (P = 0.000) whereas miR-636 were significantly higher in CRC patients (P = 0.001) (Fig. 5E,F). The ROC analysis for sensitivity was carried out using SPSS 22.0 (Fig. 5G,H). From the overall result, a double mRNA was selected as it was significantly associated with the OS.

### Functional interaction protein–protein interaction network

**Figure 6:**
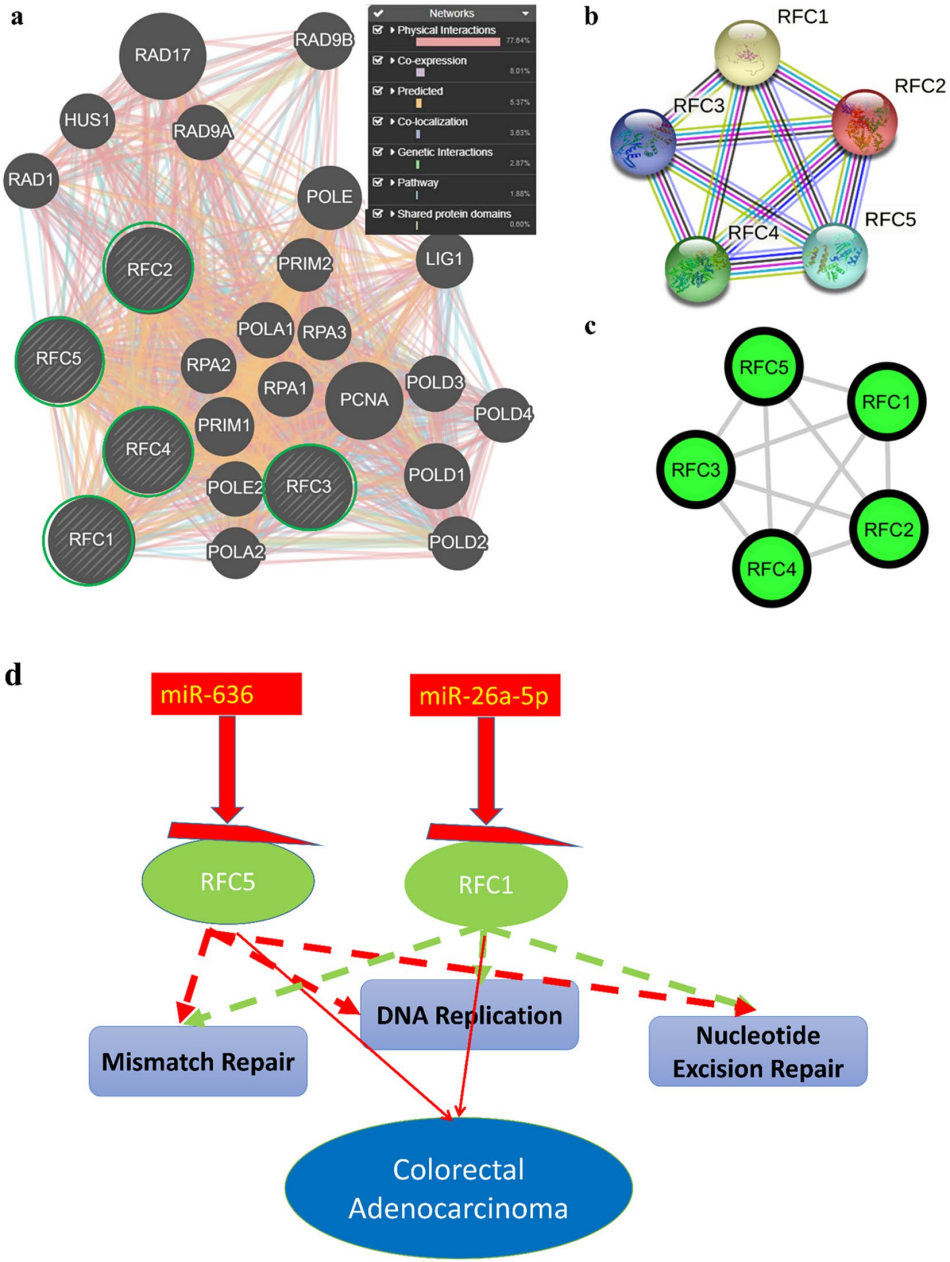
Gene interaction network (**a**) and (**b**, **c**) protein–protein interaction (PPI) network of replication factor C subunits genes (RFCs). In (**a**), input genes are indicated by stripes, with green circles representing downregulated genes in colorectal adenocarcinoma. In (**b**, **c**), PPI pairs were imported into Cytoscape software as described in "Methods and materials". Green nodes represent downregulated genes. The lines represent the interactive relationship between nodes. The highlighted DEGs represent a degree of $\ge$ 2. (**d**) Gene interaction network and pathway enrichment summary of common micro (mi)RNA targets. This schematic summary shows possible interactions of miRNAs and their colorectal adenocarcinoma targets. The red background represents upregulation and the green background indicates downregulation in colorectal adenocarcinoma, revealing significant expression in the respective validation dataset. Thick dashed red and green lines represent significant interactions with pathways. Colorectal adenocarcinoma is represented by a blue background.

We used the Gene MANIA online web tool to investigate the functional interactions of the miRNA targets, and the Reactome mismatch repair, DNA replication, and nucleotide excision repair pathway RFC’s were used to analyze the functional roles of these molecules. The analysis revealed an interaction network involving 21 other related genes, the 5 RFCs targets (*RFC1, RFC2, RFC3, RFC4, RFC5*) were entered, and a total of 1856 links were observed. Two types of interaction, physical and co-expression interaction were involved, with co-expression being the most frequent type of interaction (8.01%) among them (Fig. 6A).

Furthermore, we loaded the RFCs into the STRING database (https://string-db.org/) to extract protein–protein interaction (PPI) pairs. Subsequently, these pairs were imported into Cytoscape software to identify hub genes, as illustrated in Fig. 6B and C.

A high level of miR-26a-5p and miR-636 can suppress only a low level of *RFC1 and RFC5*, it also regulates the mismatch repair, DNA replication, and nucleotide excision repair pathway.

The elevated expression of miR-26a-5p and miR-636, leading to the targeted suppression of RFC1 and RFC5 in CRC, appears to activate the mismatch repair, DNA replication, and nucleotide excision repair signaling pathway. The interaction between these miRNAs and their target genes, along with their regulatory mechanism, is summarized in Fig. 6D.

### Association of RFC1 and RFC5 genes with immune cell infiltration

**Figure 7:**
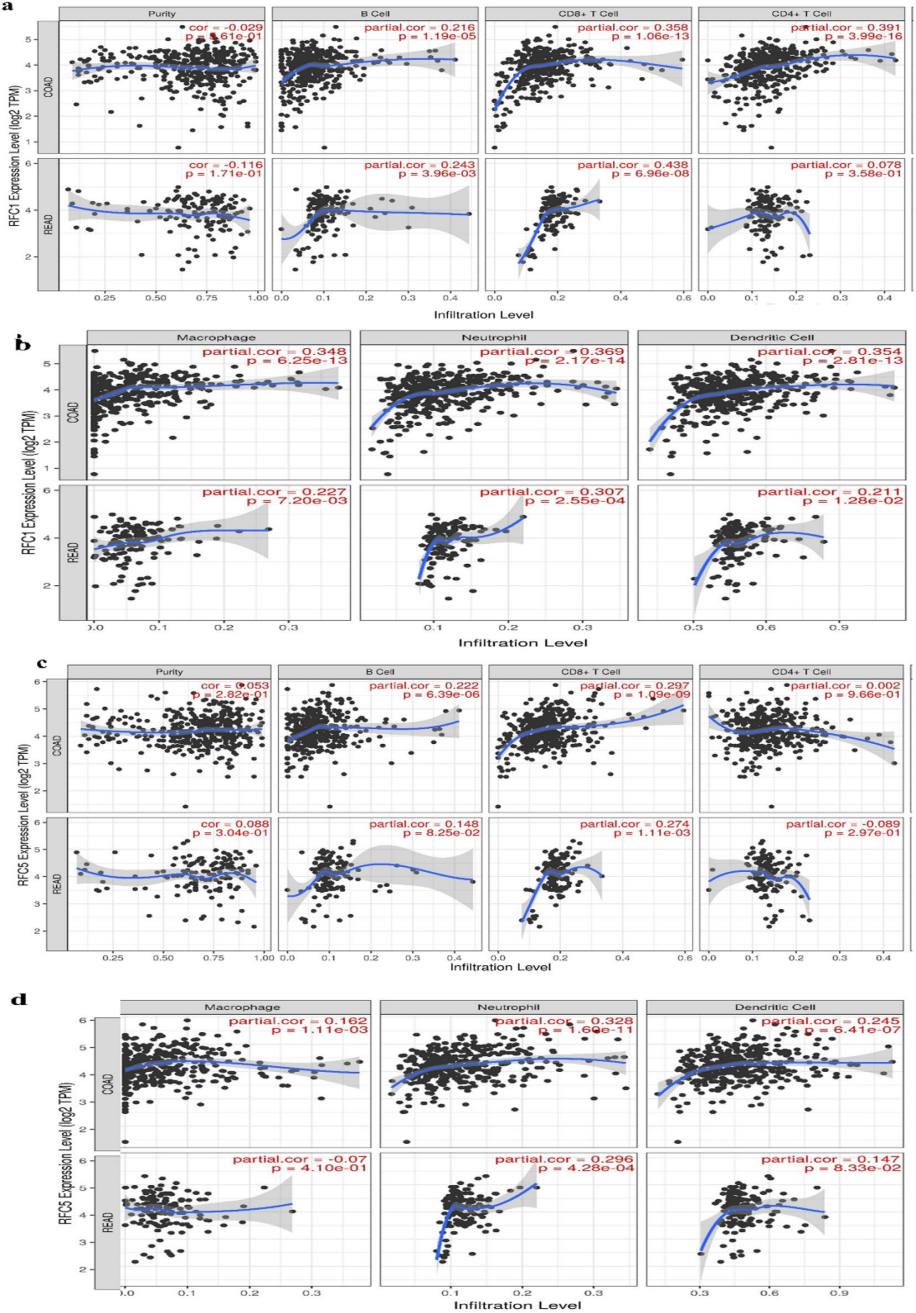
Immune filtration of the replication factor C subunit 1 (*RFC1* and* RFC5*) gene. Spearman correlations between the differentially expressed *RFC1 *and* RFC5* gene and immune cell infiltration in (**a**) colon adenocarcinoma (COAD) and (**b**) rectal adenocarcinoma (READ) patients. The TIMER web tool was used for the analysis of correlations between immune infiltration of the immune cell markers of B cells, CD4^+^ cells, CD8^+^ cells, T cells, macrophages, neutrophils, and dendritic cells vs. the *RFC1* and* RFC5* gene. Statistically, significance was accepted at *P* < 0.05 for Spearman correlations.

The relationship was examined between the *RFC1* gene with immune cell infiltration and an inflammatory response in CRC patients. The TIMER database, an online web tool, was used to predict the link between the *RFC1* and *RFC5* gene expression to immune infiltration in CRC patients (Fig. 7, Fig. S2). The results enunciated correlation between *RFC1* and *RFC5* cluster of differentiation CD4 + T cell, macrophages, and neutrophils were correlated in COAD (Colon Adenocarcinoma) and READ (Rectal Adenocarcinoma) patients.

### ERBB2, KRAS, and PTEN correlations with RFC1

**Figure 8:**
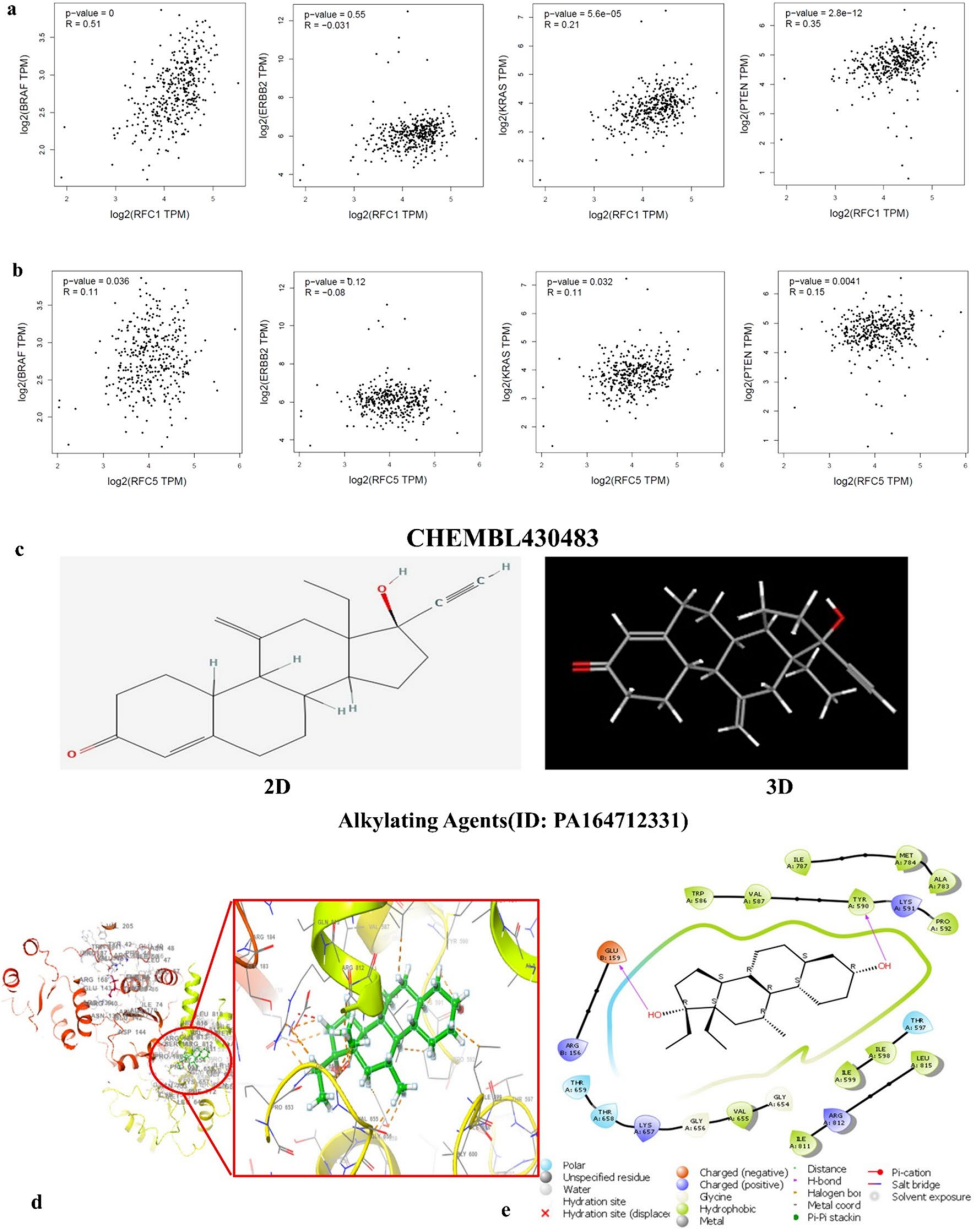
Mutation of BRAF, ERBB2, KRAS, and PTEN correlation with *RFC1* and* RFC5* (**a, b**) BRAF mutation, ERBB2 mutation, KRAS mutation and PTEN mutation. (**c**) chemical structure. (**d**) Binding of CHEMBL430483 against the RFC1 (6VVO) protein. (**e**) 2D interaction of CHEMBL430483against RFC1 (6VVO) protein.

The *RFC1* and *RFC5* correlation with the mutation of BRAF, ERBB2, KRAS, and PTEN was studied and it was found that the correlation of *RFC1* with BRAF, ERBB2, KRAS and PTEN is medium to low with R value of 0.51, − 0.031, 0.21 and 0.35 respectively. Similarly, the correlation of *RFC5* with BRAF, ERBB2, KRAS and PTEN is low with R value of 0.11, − 0.08, 0.11, 0.15 respectively (Fig. 8A).

### Small molecule predictions and docking analysis

Through the WEB-based GEne SeT AnaLysis Toolkit (WebGestalt, http://www.webgestalt.org), drug predictions were made using the single gene found by the survival analysis and HPA database. As presented by Table 2, RFC1 gene-targeted genes were finally considered to be druggable, with a p value of 1.210e-10. Compound (ID: PA16471233) (CHEMBL430483) was identified as potential small molecule (Fig. 8B).

**Table 2:** Small molecules target therapy.

Drug	Gene symbol	Gene name	P-value	FDR
Alkylating agents (ID: PA16471233)	*RFC1* *RFC2* *RFC3* *RFC4* *RFC5*	Replication factor C1-5	1.210e−10	2.224e−7

To study the interaction of the identified compound (CHEMBL430483) with the target protein docking studies were performed using Schrodinger Maestro 12.9. Two-dimensional (2D) and three-dimensional (3D) binding of CHEMBL430483 with proteins have been provided (Fig. 8C,D) and Table 3. This CHEMBL430483 has the docking score of − 7.072 with protein RFC1, the analysis of the binding pocket in the 2D interaction showed that it major binds to the pocket containing amino acid Tyr590, Pro592, Lys591, Val507, Trp586, Glu159, Arg156. Two hydrogen bond bonds are formed with amino acids Tyr590 and Glu159 which are important for interaction with the target.

**Table 3:** Identification of drug molecule using GEne SeT AnaLysis Toolkit.

S.no.	Structure	Target protein	Docking score (XP)
1	(CHEMBL430483)	RFC1	− 7.072

## Discussion

Our study revealed that *RFC1* and *RFC5* are two significant antitumor mRNA and inhibitors of tumor progression. *RFC1* and *RFC5* were downregulated in colorectal adenocarcinoma and functionally suppressed the CRC. MiR-26a-5p and miR-636 overexpression by competitively binding *RFC1 and RFC5* mRNA 3’UTR leads to mismatch repair, DNA replication, and the nucleotide excision repair signaling pathway of colorectal adenocarcinoma.

This study, used previously, published mRNA expression of RFC complex in different cancers. A computational analysis was performed by defining the RFCs correlated with miRNAs. KEGG pathway enrichment analyses were done, and a protein–protein interaction (PPI) network was performed to identify network genes. Furthermore, overall survival (OS) was obtained to determine survival biomarkers for identified colorectal cancer (CRC) patients^22^.

Replication factor C complexes play a crucial role in unloading and loading processivity clamps from DNA. They have been identified as involved in repair pathways and multiple DNA replication. The RFC (Ctf18) variant complex specifically is required to activate the intra-S-phase checkpoint at stalled replication forks and aids the establishment of sister chromatid cohesion. Unlike other RFC complexes, RFC (Ctf18) contains two non-RFC subunits, Dcc1 and Ctf8^11^. RFC1 is the largest subunit (140 kDa) of the RFC complex.

Previous studies have shown that *RFC2* the second largest subunit (40 kDa)^23^ among the RFC complexes, was upregulated and associated with some tumor tissues such as choriocarcinoma tissue and nasopharyngeal carcinoma (NPC) tissue^24,25^. Other studies have shown that high expression of *RFC2* is associated with poor survival in CRC, glioblastoma, and hepatocellular carcinoma and aids in predicting breast cancer progression and metastasis^13,26,27^. Our results revealed that *RFC2* expression is insignificant in typical vs. tumor of CRC patients and high intensity in IHA protein analysis. However, the results are inconsistent with the overall survival data of CRC patients.

The *RFC3* gene is one of the small subunits (38 kDa) of the RFC complexes has been reported preferentially blind to proliferating cell nuclear antigen (PCNA) and formed a complex. Also reports related to attenuating the *RFC3* can inhibit tumor cell proliferation are present^28^. Although the *RFC3* is a tumor suppressor gene, it has been associated with poor survival in triple-negative breast cancer, ovarian tumor, lung adenocarcinoma, esophageal adenocarcinoma, hepatocellular carcinoma, suggesting that *RFC3* may be a potential risk oncogenic gene involved in tumorigenesis^29–34^. In our results, *RFC3* expression is insignificant in typical vs. tumor of CRC patients but the results are inconsistent with overall survival data of CRC patients.

The replication factor C subunit 4 (*RFC4*) has been reported to be involved in DNA replication as a clamp loader of PCNA^35^. The *RFC4* has also been identified in previous studies as a tumor suppressor gene and has been associated with poor prognosis in CRC, HCC, cervical cancer, oral tongue squamous cell carcinoma, NSCLC, and esophageal squamous cell carcinoma^36–41^. In our results, *RFC4* expression was significant in typical vs. tumor tissue of CRC patients but showed inconsistent results with overall survival data of CRC patients.

Overall, *RFC5* (36 kDa) is a tumor suppressor and indicates poor survival in many cancers such as lung cancer and glioblastoma^42–44^. In our results, *RFC5* expression was significant in normal vs. tumor tissue of CRC patients, and showed high intensity with IHA protein analysis, and consistent results with overall survival data of CRC patients. However, *RFC5 is targeted* with associated miR-636 which is why we selected *RFC1 and RFC5* as a prognostic marker for colorectal adenocarcinoma.

We explored the potential mechanisms of *RFC1* and *RFC5* that mediated colorectal adenocarcinoma by focusing on potential microRNA. Here we identified that high expression of miR-26a-5p and miR-636 were associated with a poor prognosis of CRC patients. Recently, microRNAs have become famous for cancer treatment however, some previous studies identified, upregulated miR-26a-5p to be a tumor suppressor oncomir in CRC and sponge or a mediating oncomir that regulated autophagy, cell migration, cell proliferation, and invasion via the PI3K-AKT pathway in CRC^18,45–51^**.** miR-636 was identified as an onco-suppressor in lung cancer, nasopharyngeal carcinoma, cervical cancer, endometrial cancer, ovarian cancer, hepatocellular carcinoma^19,52–61^. High expression of miR-26a-5p and miR-636 has been correlated with poor OS of CRC patients, which was found consistent with our results.

The tumor microenvironment plays a critical role in the cancer progression of metastatic cancer, and tumor-associated macrophages (TAMs) form essential components of the tumor microenvironment. High TAMs is associated with invasion, migration, and IL6 for tumor progression of CRC metastasis^62^. Tumor infiltration is associated with six cells B cells, CD8+, CD4+, macrophages, neutrophils, and dendritic cells^63^. Our results showed that the *RFC1* and *RFC5* are associated with CD8+, CD4+, neutrophils, and macrophages. Which means the *RFC1* and *RFC5* can be used as a prognostic tumor marker for colorectal adenocarcinoma.

Our research is first to show a link between the *RFC1* and *RFC5* with tumor prognosis. This study showed that *RFC1* and *RFC5* is associated with overall survival and the prognosis of CRC in patients. Some previous studies also showed that the miR-26a-5p and miR-636 targeted *RFC1* and *RFC5* in the mismatch repair, DNA replication, nucleotide excision repair pathway, mediated colorectal adenocarcinoma, and our results are consistent with those of a previous study. Moreover, we also predicted a small molecule, CHEMBL430483 targeted RFC1 through webGestalt analysis. The interaction assessments of CHEMBL430483 docked positions, supports that it can be developed as potent inhibitor.

Nevertheless, some limitations exist in our study. It was difficult to collect sufficient CRC patient samples and to carry out the in vitro and in vivo studies or find a suitable public database to evaluate the clinical significance of miR-26a-5p or targeted *RFC1* and miR-636 targeted *RFC5* in terms of expression levels and CRC progression.

As differential expression miRNA data was limited, we only used twenty miRNAs in this investigation, leading to only two miR-26a-5p and miR-636 being identified as linked with CRC patients.

In the future, the tumor-suppressive role of miR-26a-5p targeted *RFC1* and miR-636 targeted *RFC5* in CRC progression needs to be further investigated in a larger cohort of patients.

We believe that miR-26a-5p targeted *RFC1* and miR-636 targeted *RFC5* in the mismatch repair, DNA replication, and nucleotide excision repair pathway, play an essential role by mediating colorectal adenocarcinoma progression.

## Materials and methods

### Data collection

The mRNA sequencing data, molecular categories, and clinical information of colorectal cancer patients, as well as other cancer types, were sourced from the TCGA, COAD, and READ databases (https://tcga-data.nci.nih.gov/, with links to COAD and READ accessed on 24 December 2021). Immunohistochemistry (IHC) staining data were retrieved from the Human Protein Atlas, available at https://www.proteinatlas.org/ (accessed on 24 December 2021), and GEPIA, available at http://gepia.cancer-pku.cn/index.html (accessed on 24 December 2021), respectively. The expression patterns of normal colon and tumor tissues were obtained from the TCGA database. The STRING database, accessible at https://www.string-db.org/ (accessed on 24 December 2021), and Cytoscape software (version 3.4.0, http://www.cytoscape.org), were utilized to construct the protein–protein interaction (PPI) network and enrichment pathway. The immune cell content file of the TCGA samples was acquired from the TIMER database (https://cistrome.shinyapps.io/timer/, accessed on 24 December 2021).

### Expression analysis of RFCs in CRC

The CCLE dataset (https://portals.broadinstitute.org/ccle, accessed on 28 December 2021) was employed to illustrate the mRNA expression levels of RFCs in cancer cell lines. The expression data are presented in a heatmap using the CIMminer web tools. Additionally, this study utilized the GEPIA databases, accessible at http://gepia.cancer-pku.cn/index.html, to examine the expression levels of RFCs in both normal and CRC tissues. The analysis applied a threshold of an absolute log base 2 of the fold change (Log2FC) set to 1, and the q value set to 0.05^64^.

### Validation of RFCs genes in CRC patients

To validate the role of RFC genes, we conducted a survival analysis using the GEPIA database, which is accessible at http://gepia.cancer-pku.cn/detail.php?gene=&clicktag=survival. The Kaplan–Meier survival analysis graph was generated for a selected cohort of 362 CRC patients with both mutation and RNA sequence data. Additionally, we predicted the survival of miRNA using a specific cohort from GSE29623. The overall analysis was performed using the statistical software SPSS version 22.0 (SPSS, Chicago, IL, USA, www-01.ibm.com).

### Pathway and enrichment analysis

The Database for Annotations, Visualization, and Integrated Discovery (DAVID bioinformatics, available at https://david.ncifcrf.gov/), was utilized to differentiate the expression genes based on their cellular components, molecular functions, and biological processes, utilizing resources from the Gene Ontology knowledgebase (GO, available at http://www.geneontology.org/)^65^. DAVID was employed for the enrichment analysis of RFC genes, and the pathway analysis was conducted with reference to the Kyoto Encyclopedia of Genes and Genomes (KEGG, http://www.genome.jp/kegg/) database, using FDR < 0.25 as the cutoff point^66^.

### Protein expression analysis of RFCs in CRC

The intensity of RFC proteins in CRC tissues within the human body was examined using the Human Protein Atlas (HPA) database, accessible at https://www.proteinatlas.org/^67^. The HPA database offers over 700 antibodies to human proteins for matching with 400,000 high-resolution images. The following formula was employed to assess each intensity and fraction combination, automatically converting it into a protein expression level score. The scores were categorized as follows: negative—not detected; weak—not detected; weak combined with either 25–75 percent or 75 percent—low; moderate—low; moderate combined with either 25–75 percent or 75 percent—medium; strong—medium; strong combined with either 25–75 percent or 75 percent—high.

### Construction of protein–protein interaction network

A protein–protein interaction network was constructed using the online web tool 'STRING' (http://www.string-db.org/)^68^. This tool provides information on known and predicted protein interactions, derived from four sources: genomic context, co-expression, high-throughput experiments, and previous knowledge. A score of 0.4 (medium confidence) was selected as the cutoff criterion. PPI pairs were then analyzed using Cytoscape software (version 3.4.0, http://www.cytoscape.org) with the CytoNCA app. Hub genes, representing highly connected genes, were identified by calculating the degree value (the number of lines connecting the genes) with a cutoff of ≥ 2.

### Prediction of microRNA for RFCs

MiRNAs were predicted using the GSE29623 cohort study, encompassing 143 miRNA samples in colon adenocarcinomas, and miRWalk websites for 1047 miRNAs of RFC1 and 765 miRNAs of RFC5 3’UTR. A target score of < 0.70 was selected in the miRWalk webtools. Additionally, a Venn diagram was employed to illustrate common miRNAs and their shared gene targets^69^.

### Clinical examination of the microRNA

For the clinical examination of differentially expressed (DE) miRNAs, a survival analysis was conducted using CRC patients. The CRC metabase for tissue expression of miRNA, including GSE29623 with a total of 65 cases and GSE126093 with 20 cases, was selected. Expression profiles were compared based on low or high expression using the Mann–Whitney U test. Statistical analysis was performed using SPSS version 22.0 (SPSS, Chicago, IL, USA, www-01.ibm.com) for plotting Kaplan–Meier curves, boxplots, and ROC curves. A P-value < 0.05 was considered statistically significant.

### Clinical relevance of mutation of BRAF, ERBB2, KRAS, and PTEN

GEPIA, available at http://gepia.cancer-pku.cn/index.html (accessed on 19 September 2022), was utilized to examine the correlation of mutations with RFC1 and RFC5.

### Immune infiltration analysis RFCs in CRC

The TIMER database (available at http://timer.cistrome.org/; accessed on 24 December 2021) was employed for the analysis of immune infiltration. As a comprehensive database offering analysis of immune infiltrates in various cancer types^70,71^, it was used in this study to investigate the involvement of RFCs in immune infiltrates in CRC. Scatterplots were used to present the relationship between gene expression and estimated infiltrate values, with the level of significance set at P < 0.05.

### Small-molecule drug-targeting therapy for DEGs

The drugs linked to DEGs were predicted using the web-based GEne SeT AnaLysis Toolkit (WebGestalt, http://www.webgestalt.org), an integrated system for gene analysis^72^.

### Docking study

Molecular docking studies were validated to confirm the selected drug to study the interaction of drug with the protein. For docking investigations, the 3D structures of the target proteins (PBD ID-6VVO) was acquired from the Protein Data Bank (PDB) at (https://www.rcsb.org/)^73^. Compound (ID: PA16471233) (CHEMBL ID 430483) identified from webGestalt was downloaded from the PubChem databases (https://pubchem.ncbi.nlm.nih.gov/)^74^. The molecular docking software, maestro v12.8 (Schrödinger), was used to perform molecular docking and visualize interactions^75^.

## Conclusions

Our study concluded that high expression of miR-26a-5p targeting *RFC1* and miR-636 targeting *RFC5* expression in the mismatch repair, DNA replication, nucleotide excision repair pathway is expected to be a potential biomarker for detecting CRC at an early stage and small molecule CHEMBL430483 can be developed for the treatment of the disease. Our findings may provide novel directions and strategies for CRC therapies.

### Supplementary Information

Supplementary Information.

## Data Availability

The original contributions presented in the study are included in the article, further inquiries can be directed to the corresponding authors.

## Abbreviations

RFCs: Replication factor C
CRC: Colorectal cancer
CCLE: Cancer cell line encyclopedia
COAD: Colon adenocarcinoma
READ: Rectal adenocarcinoma
GEPIA: Gene expression profiling interactive analysis
TIMER: Tumor immune estimation resources
OS: Overall survival
STRING: Search tool for the retrieval of interacting genes/proteins
