# Supplementary material for: Identification of expression profiles and prognostic value of RFCs in colorectal cancer
Source: Sci Rep. 2024 Mar 19;14:6607. doi: 10.1038/s41598-024-56361-2 (PMC10951252; doi:10.1038/s41598-024-56361-2)
Supplement: Supplementary file 1 — Supplementary Information. [file 41598_2024_56361_MOESM1_ESM.docx]

**Supplementary Information**

**Identification of Expression Profiles and Prognostic Value of RFCs in Colorectal Cancer**

**Supplementary Tables**

**Table S1: List of miRNA’s targeted genes**

| **Gene symbol** | **No. of targets** | **List of miRNAs** |
| --- | --- | --- |
| RFC1 | 32 | hsa-miR-7159-5p, hsa-miR-5692a, hsa-miR-4668-5p, hsa-miR-30a-5p, hsa-miR-12122, hsa-miR-3692-3p, hsa-miR-3680-3p, hsa-miR-378f, hsa-miR-548v, hsa-miR-378i, hsa-miR-4762-5p, hsa-miR-422a, hsa-miR-30b-5p, hsa-miR-1257, hsa-miR-378a-3p, hsa-miR-128-3p, hsa-miR-30e-5p, hsa-miR-378b, hsa-miR-9985, hsa-miR-520d-5p, hsa-miR-373-3p, hsa-miR-378c, hsa-miR-216a-3p, hsa-miR-378e, hsa-miR-3135a, hsa-miR-5087, hsa-miR-6783-5p, hsa-miR-378h, hsa-miR-494-3p, hsa-miR-26a-5p, hsa-miR-181a-5p, hsa-miR-187-5p |
| RFC5 | 20 | hsa-miR-30d-5p, hsa-miR-7-2-3p, hsa-miR-5692a, hsa-miR-885-5p, hsa-miR-6867-5p, hsa-miR-30c-5p, hsa-miR-30a-5p, hsa-miR-12122, hsa-miR-3692-3p, hsa-miR-5011-5p, hsa-miR-6504-3p, hsa-miR-4638-3p, hsa-miR-221-3p, hsa-miR-5681b, hsa-miR-636, hsa-miR-520e-3p, hsa-miR-30e-5p, hsa-miR-3129-3p, hsa-miR-147b-5p, hsa-miR-1251-3p |

**Table S2 List of survival-related microRNAs**

| **Gene Name** | **microRNA name** | **Overall Survival (P value)** | **Disease free survival (P value)** |
| --- | --- | --- | --- |
|  |  |  |  |
| **RFC1** | hsa-miR-181a-5p | 0.417 | 0.593 |
|  | hsa-miR-30a-5p | 0.110 | 0.817 |
|  | hsa-miR-378f | 0.278 | 0.291 |
|  | hsa-miR-378i | 0.278 | 0.291 |
|  | hsa-miR-422a | 0.130 | 0.071 |
|  | hsa-miR-30b-5p | 0.376 | 0.815 |
|  | hsa-miR-378a-3p | 0.278 | 0.291 |
|  | hsa-miR-128-3p | 0.117 | 0.475 |
|  | hsa-miR-30e-5p | 0.081 | 0.722 |
|  | hsa-miR-378b | 0.278 | 0.291 |
|  | hsa-miR-378c | 0.278 | 0.291 |
|  | hsa-miR-378h | 0.278 | 0.291 |
|  | hsa-miR-494-3p | 0.075 | 0.239 |
|  | hsa-miR-26a-5p | **0.017** | 0.351 |
| **RFC5** | hsa-miR-30d-5p | 0.124 | 0.978 |
|  | hsa-miR-30c-5p | 0.247 | 0.647 |
|  | hsa-miR-30a-5p | 0.110 | 0.817 |
|  | hsa-miR-221-3p | 0.895 | 0.133 |
|  | hsa-miR-636 | **0.050** | 0.739 |
|  | hsa-miR-30e-5p | 0.081 | 0.722 |

**Bold p ≤ 0.05 is significant**

**Supplementary Figures**

**
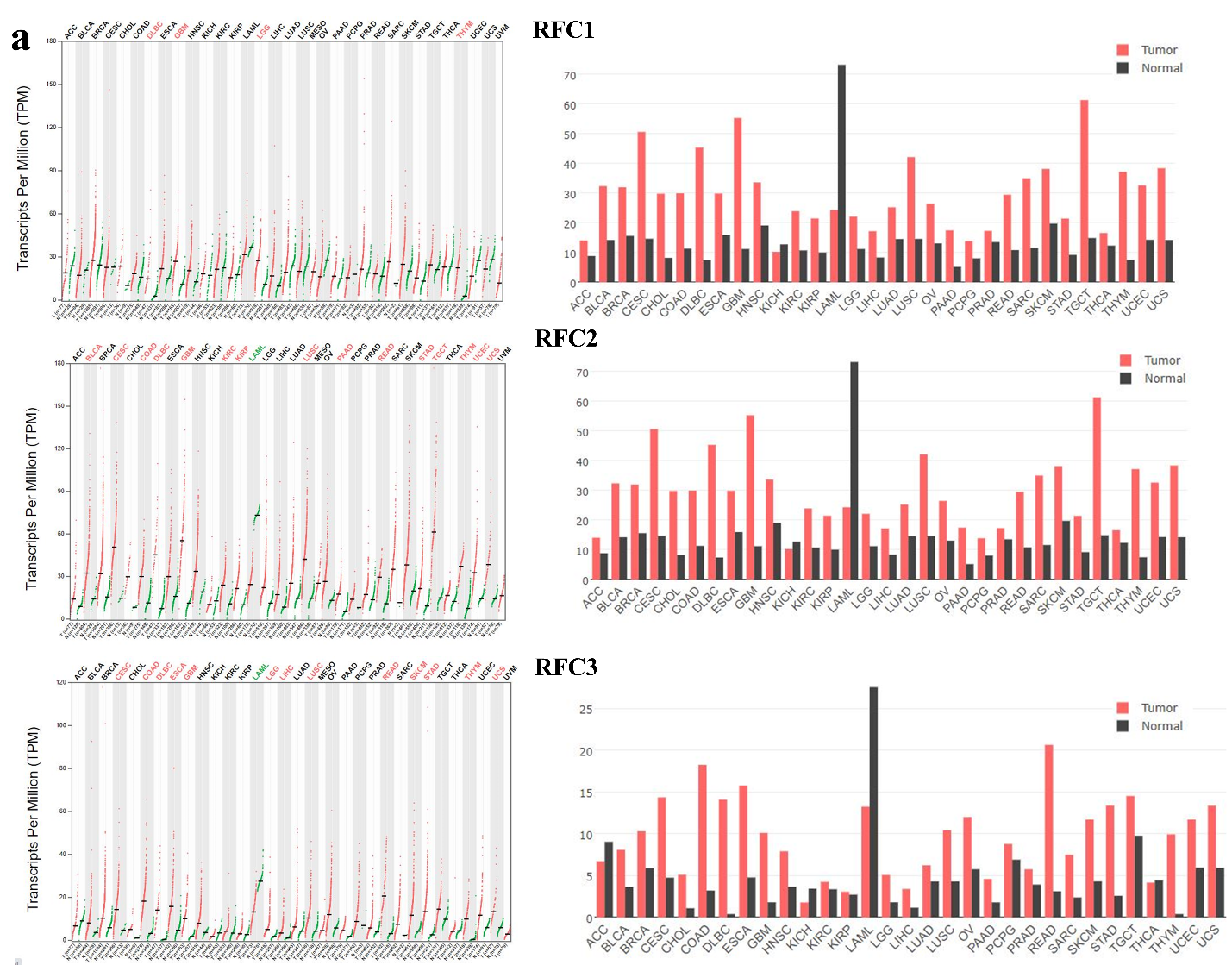
**

**
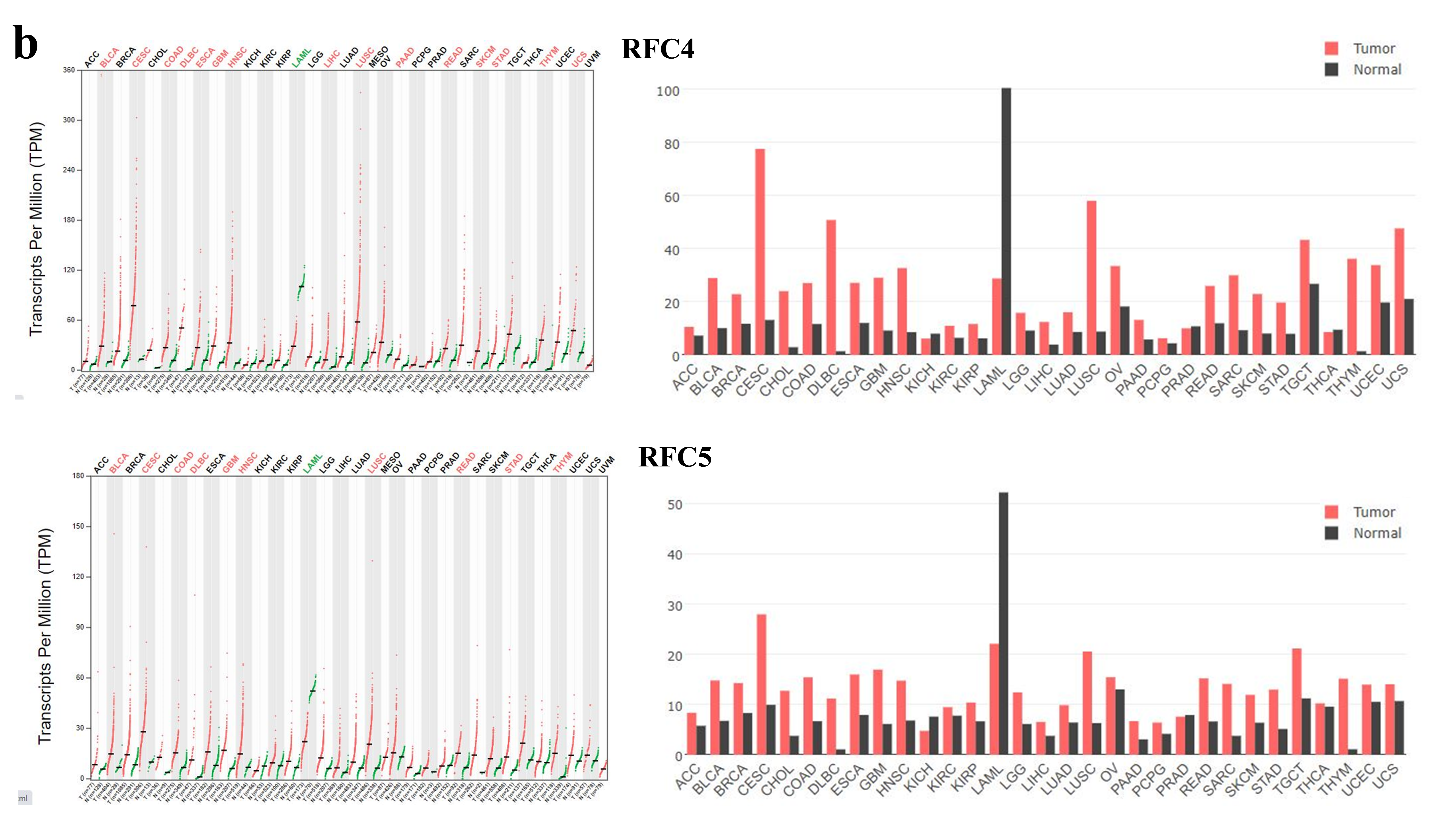
**

**Figure S1. The gene expression profile of RFCs (A) *RFC1, RFC2, RFC3*, (B) *RFC4, RFC5*.** These were evaluated in 27 TCGA tumor samples vs. normal tissues using the GEPIA web tool. The black bar indicates normal tissues, while the red bar indicates expressions of RFCs in tumor tissues. Each GTEx normal data point (green) and its matched TCGA tumor (red) used TPM (transcripts per million (log2 (TPM + 1)). X-axis: number of tumors and normal samples. ACC (adrenocortical carcinoma), BLCA (bladder urothelial carcinoma), BRCA (breast invasive carcinoma), CESC (cervical squamous cell carcinoma and endocervical adenocarcinoma), CHOL (cholangiocarcinoma), COAD (colon adenocarcinoma), DLBC (lymphoid neoplasm diffuse large B-cell lymphoma), ESCA (esophageal carcinoma), GBM (glioblastoma multiforme), HNSC (head and neck squamous cell carcinoma), KICH (kidney chromophobe), KIRC (kidney renal clear cell carcinoma), KIRP (kidney renal papillary cell carcinoma), LAML (acute myeloid leukemia), LGG (brain lower-grade glioma), LIHC (liver hepatocellular carcinoma), LUAD (lung adenocarcinoma), LUSC (lung squamous cell carcinoma), MESO (mesothelioma), OV (ovarian serous cystadenocarcinoma), PAAD (pancreatic adenocarcinoma), PCPG (pheochromocytoma and paraganglioma), PRAD (prostate adenocarcinoma), READ (rectum adenocarcinoma), SARC (sarcoma), SKCM (skin cutaneous melanoma), STAD (stomach adenocarcinoma), TGCT (testicular germ cell tumors), THCA (thyroid carcinoma), THYM (thymoma), UCEC (uterine corpus endometrial carcinoma), UCS (uterine carcinosarcoma).

**
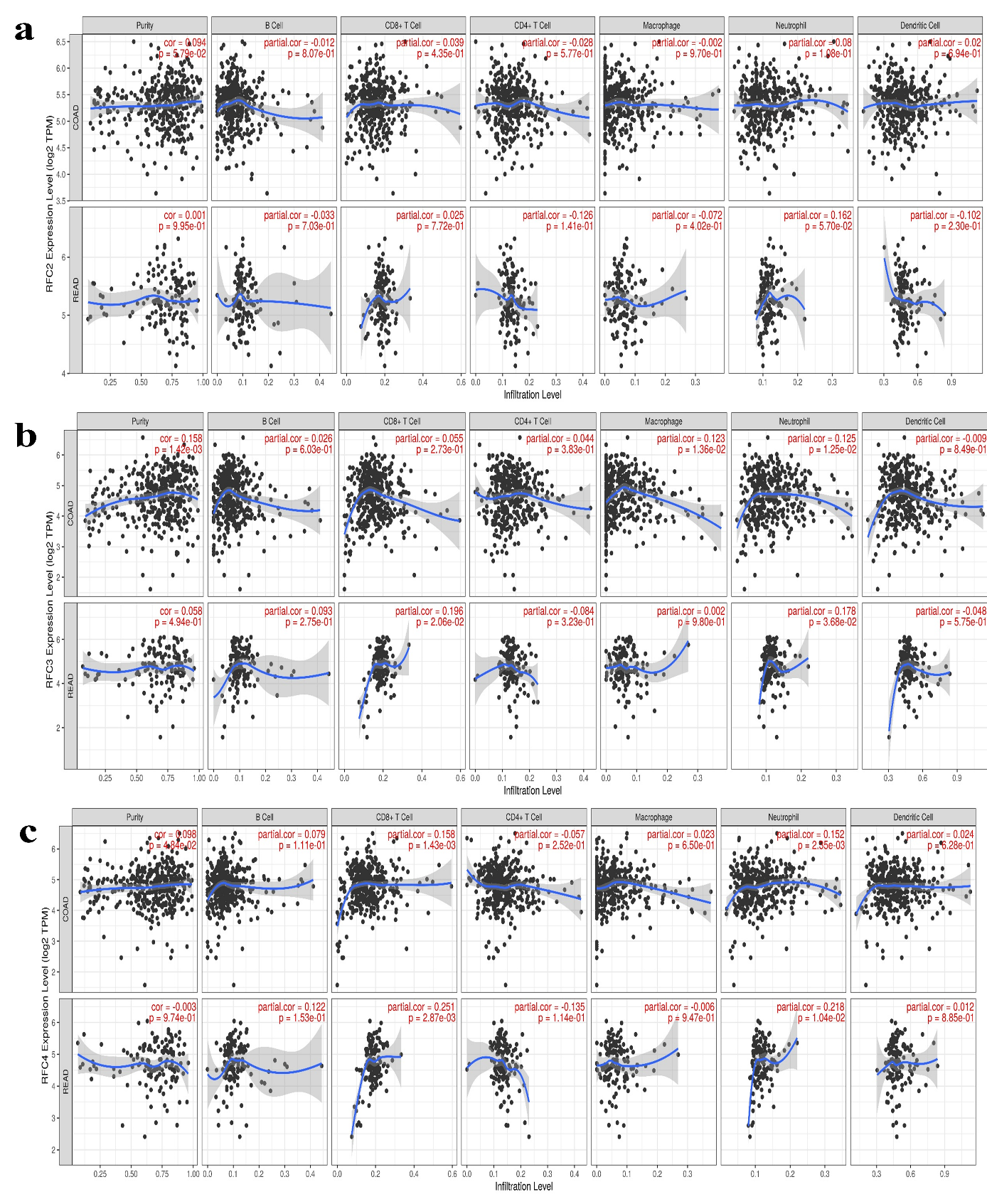
**

**Figure S2. Correlations between differentially expressed *RFC2, RFC3, and RFC4*, genes and immune cell infiltration in primary colorectal cancer (CRC) patients (A-C) (TIMER).**
